# Viral infections of the central nervous system increase the risk of knee osteoarthritis: a two-sample mendelian randomization study

**DOI:** 10.1007/s40520-025-02927-7

**Published:** 2025-01-21

**Authors:** Mingyi Yang, Yani Su, Ke Xu, Pengfei Wen, Jiale Xie, Xianjie Wan, Wensen Jing, Zhi Yang, Lin Liu, Peng Xu

**Affiliations:** 1 Department of Joint Surgery, HongHui Hospital, Xi’an Jiaotong University, Xi’an, Shaanxi 710054 China; 2 Xi’an Key Laboratory of Pathogenesis and Precision Treatment of Arthritis, Xi’an, Shaanxi 710054 China

**Keywords:** Central nervous system, Osteoarthritis, Causal, Genetic

## Abstract

**Objective:**

Osteoarthritis (OA) represents a condition under the influence of central nervous system (CNS) regulatory mechanisms. This investigation aims to examine the causal association between viral infections of the central nervous system (VICNS) and inflammatory diseases of the central nervous system (IDCNS) and knee osteoarthritis (KOA) at the genetic level.

**Methods:**

In this investigation, VICNS and IDCNS were considered as primary exposure variables, while KOA served as the primary outcome. Employing a two-sample mendelian randomization (MR) approach, we conducted an analysis utilizing summary data derived from genome-wide association studies (GWAS). The GWAS summary data pertaining to VICNS and IDCNS were procured from the Finnish consortium, whereas the IEU OpenGWAS database furnished the requisite data for KOA. To ensure the robustness of our genetic causal assessment, a comprehensive array of sensitivity analyses was undertaken, encompassing evaluations of heterogeneity, horizontal pleiotropy, outlier identification, leave-one-out analyses, and assessment of the normal distribution.

**Results:**

The results of the MR analyses revealed a suggestive positive genetic causal relationship between VICNS and KOA (*P* = 0.012, odds ratio [OR] with a 95% confidence interval [CI] of 1.033 [1.007–1.059]). Conversely, the MR analyses did not indicate any evidence of genetic causation between IDCNS and KOA (*P* = 0.575, OR 95% CI = 0.986 [0.940–1.035]). Importantly, the genetic causal assessment of the exposure and outcome variables did not demonstrate any indications of heterogeneity, horizontal pleiotropy, or outliers. Furthermore, this assessment remained robust against the influence of individual single nucleotide polymorphisms (SNPs) and exhibited adherence to a normal distribution.

**Conclusion:**

The result of this study has elucidated a suggestive positive genetic causal link between the VICNS and KOA. However, no such genetic causal relationship was observed between the IDCNS and KOA. These findings substantiate the genetic underpinnings supporting the association between the CNS and OA.

**Supplementary Information:**

The online version contains supplementary material available at 10.1007/s40520-025-02927-7.

## Introduction

Osteoarthritis (OA) represents the predominant chronic joint ailment and is recognized as an age-related degenerative disease. Although OA can impact various joints of differing sizes throughout the body, the knee joint stands as the most commonly affected site. Approximately one in eight individuals aged 60 and above experience symptomatic knee OA (KOA) [1], hence drawing substantial attention within the sphere of OA research. As a prevalent degenerative joint disorder worldwide, OA constitutes a comprehensive joint pathology characterized by the gradual deterioration of articular cartilage, synovial inflammation, osteophyte formation, and subchondral osteosclerosis, primarily manifesting as chronic pain [2]. Ranking as the tenth most influential contributor to global disability, the prevalence of OA has escalated in tandem with an aging demographic. Notably, OA stands as a principal source of disability among the elderly and significantly impairs mobility within the United States [3]. Presently, effective treatments for OA are limited, lacking treatments to impede its progression. Clinical management chiefly centers on symptom alleviation, with advanced OA often necessitating joint replacement surgery. The impact of OA extends beyond the individual’s health, generating substantial medical expenses and imposing a significant societal burden. Consequently, there exists an urgent need for extensive research into OA to decelerate disease advancement and diminish the incidence of end-stage joint replacement, thereby alleviating both individual and societal burdens.

Pain represents the predominant symptomatic manifestation throughout the progression of OA. While the degradation of cartilage traditionally serves as a hallmark indicative of OA’s advancement, the correlation between the extent of cartilage degradation and the severity of pain appears to be tenuous. Conversely, alterations observed in bone structure and synovial tissue exhibit a marginally stronger association with the degree of pain experienced by patients [4]. The degenerative modifications occurring in OA, involving cartilage, bone, and synovium, has been characterized as joint organ diseases, wherein a balance exists between intra-articular anabolic and catabolic changes [5]. This framework partly confines the exploration of OA-induced pain to the localized joint context. However, ongoing investigations into OA increasingly acknowledge that the physiological alterations instigated by OA-related pain transcend joint boundaries, as repetitive pain signaling induces adaptations within the neural pathways of the spinal cord and brain [4]. Research indicates that the pain stemming from OA is influenced by multifaceted processes of varying degrees. Notably, local inflammatory factors like synovitis constitute a crucial factor in eliciting joint pain in OA. Additionally, general factors encompassing metabolic shifts, diabetes, genetic predispositions, and psychological elements may exacerbate OA-related pain. Moreover, neuroplastic changes within the nociceptive system, involving both peripheral and central sensitization, contribute significantly to the modulation of OA-induced pain [6].

Patients diagnosed with OA are predisposed to the dysregulation of various central feedback mechanisms governing sympathetic tone, inflammation, circadian rhythms encompassing both central and peripheral clocks, gut microbiota, metabolic redox, and the overall pathology of affected joints [7]. Notably, recent attention has been directed toward understanding the intricate association between OA and the central nervous system (CNS). Investigations have indicated that the experience of OA-related pain is, in part, attributed to CNS processes, with identified irregularities in the processing of nociceptive stimuli within the CNS that bear resemblance to those observed in other chronic pain conditions [8]. Current research underscores the crucial role of the nervous system in regulating skeletal metabolism, highlighting the significance of skeletal interoception in maintaining bone homeostasis [9]. The CNS emerges as a pivotal regulator of the skeletal system, exhibiting robust sensory and sympathetic innervation vital for bone homeostasis and pain modulation [10–13]. Bones are extensively innervated sensorily, and the interaction between the CNS and bone physiology has been elucidated through viral tracer experiments and immunofluorescence, elucidating the pathway from bone sensory neurons to the central nervous system [14, 15]. It is increasingly evident that OA transcends local joint affliction, with the CNS exerting a significant influence on its progression. Consequently, exploring the regulatory impact of the CNS on OA represents a promising avenue for research to mitigate disease progression. Therefore, further investigation into the multifaceted relationship between the CNS and OA across various levels is imperative to inform clinical decision-making in managing OA.

The advancement of GWAS has facilitated significant strides in comprehending the genetic underpinnings of various diseases. A proliferation of genetic analysis techniques has emerged for the examination of diseases or traits. Among these methodologies, Mendelian randomization (MR) analyses have gained widespread adoption as a statistical tool to infer causal relationships in disease genetics research. MR analyses commonly employ single nucleotide polymorphisms (SNPs) as instrumental variables (IVs) to investigate the causal association between an exposure and an outcome. This analytical approach is adept at mitigating confounding factors and reversing causation, thereby yielding robust estimates. A recent comprehensive proteomic MR study delved into potential therapeutic targets for prevalent cancers [16]. Moreover, MR analysis was used to explore the correlation between resting heart rate and atrial fibrillation [17]. Motivated by the conjecture that CNS damage might impact OA progression, a two-sample MR study was conducted to scrutinize the potential causal linkage between viral infections of the central nervous system (VICNS) and inflammatory diseases of the central nervous system (IDCNS) with KOA. Our study adheres to the STROBE-MR guidelines for the reporting of MR, the STROBE-MR checklist of our study is shown in Supplementary Table 1.

## Materials and methods

### Study design

In this investigation examining the genetic causal relationship between VICNS and IDCNS as exposures and KOA as outcomes, a two-sample MR analysis was conducted. The analysis adhered rigorously to the fundamental assumptions of MR methodology, comprising: (1) the IVs exhibited a robust association with the exposures (VICNS and IDCNS); (2) the IVs remained independent of both the outcome (KOA) and potential confounding variables; and (3) the effects of the IVs on the outcomes were solely mediated through the exposures. GWAS summary data concerning both exposures and outcomes in European populations were sourced from publicly available databases for analysis. Consequently, ethical statements and informed consent were deemed unnecessary for this study. A detailed exposition of the data utilized in this investigation can be found in Supplementary Table 2.

### Data source

We acquired the GWAS summary data for two distinct exposures, VICNS and IDCNS, from the Finnish consortium, accessible at https://www.finngen.fi/. The VICNS GWAS summary data encompasses 1,155 cases and 217,637 controls, incorporating a total of 16,380,466 SNPs. Similarly, the IDCNS GWAS summary data includes 1,307 cases and 217,485 controls, including 16,380,466 SNPs. All cases were identified through the application of the M13 code in the International Classification of Diseases-Tenth Revision (ICD-10). Genotyping procedures involved the utilization of Illumina and Affymetrix chip arrays (Illumina Inc, San Diego, and Thermo Fisher Scientific, Santa Clara, CA, USA, respectively). For more comprehensive details regarding the utilized data, interested parties are encouraged to refer to the FinnGen website. Additionally, the IEU OpenGWAS database (https://gwas.mrcieu.ac.uk/) contains GWAS summary data concerning the outcome known as KOA. This dataset comprises 24,955 cases and 378,169 controls, examining 29,999,696 SNPs. The diagnosis of KOA was based on hospital records using the ICD10 code “HES_p_M17_BIN_Gonarthrosis.” Participants with any pre-existing musculoskeletal disorders or relevant symptoms such as inflammatory polyarthropathies (e.g., gout, rheumatoid arthritis, juvenile arthritis, and other arthropathies) were excluded from this analysis. In summary, the samples underwent genotyping with the UK Biobank Axiom array (Affymetrix).

### IVs selection

The IVs utilized in the MR analysis underwent a comprehensive selection process characterized by rigorous screening protocols. Initially, SNPs highly correlated with the exposure variables VICNS and IDCNS were acquired, employing stringent criteria for correlation strength, set at a significance threshold of *P* < 5 × 10^− 8^ and an F statistic > 10. The calculation of the F statistic was conducted according to the formula: F = R^2^(N-K-1)/K(1-R^2^). Notably, in instances where an adequate quantity of SNPs meeting the *P* < 5 × 10^− 8^ criterion for MR analysis could not be secured, an adjusted threshold of *P* < 5 × 10^− 6^ was permissible based on prior literature [18–20]. SNPs possessing a minor allele frequency (MAF) < 0.01 were systematically eliminated from consideration [21]. Subsequent refinement involved the selection of SNPs demonstrating limited linkage disequilibrium (LD), with LD r^2^ values < 0.001 and a clump distance exceeding 10,000 kb, serving as IVs. Furthermore, IVs exhibiting associations with the outcome variable (KOA) were excluded, adhering to the correlation threshold consistent with that employed for SNPs and exposure. Mitigation against confounding factors was executed by utilizing the PhenoScanner database (http://www.phenoscanner.medschl.cam.ac.uk/) to identify SNPs linked to potential confounders, notably encompassing factors such as advanced age and obesity [22–24]. Additionally, SNPs characterized by palindromic sequences and intermediate allele frequencies were systematically omitted from the analytical pipeline.

### MR analysis

We employed a diverse array of MR assays to establish the genetic underpinnings of the relationship between exposure factors (VICNS and IDCNS) and the resultant outcome (KOA). Specifically, our study incorporated a comprehensive suite of eight distinct MR analysis methodologies. The primary method utilized for evaluating causal relationships was the random-effects inverse variance weighted (IVW) approach, recognized for its central role in causal assessment. Furthermore, we conducted assessments employing four additional MR methodologies—namely, MR Egger, weighted median, simple mode, and weighted mode—to examine the genetic causality between the aforementioned exposures and the development of KOA. Significantly, we also employed three supplementary MR analyses, designated as maximum likelihood, penalised weighted median, and fixed effects IVW, to corroborate the genetic causal inferences. Among the gamut of eight MR analysis methodologies employed, the random-effects IVW method emerged with the highest statistical power and proficiency in evaluating causality, thereby establishing it as the gold standard for genetic causality assessment in MR analyses. The IVW approach hinges on the assumption that all genetic variants serve as valid IVs and leverages meta-analysis principles to amalgamate diverse SNPs to ascertain the Wald ratio of causality. Consequently, this methodology furnishes a coherent genetic causal appraisal between exposure factors and the resulting outcome. Notably, in scenarios devoid of horizontal pleiotropy, IVW methods have demonstrated consistent and accurate causal assessments, as evidenced in prior research [25].

### Sensitivity analysis

The sensitivity analysis of MR findings constitutes a crucial facet in the evaluation of genetic causal inference, serving to validate the accuracy and ensure the dependability of research outcomes. In this study, a comprehensive suite of sensitivity analyses was undertaken to ascertain the robustness of the MR analysis. Initially, we assessed the heterogeneity within the MR analysis employing two distinct methodologies: Cochran’s Q statistic applied to the MR-IVW approach, and Rucker’s Q statistic utilized for MR Egger. Establishing the absence of horizontal pleiotropy assumes particular significance as a fundamental precondition for the credibility of the random effects IVW analysis. To examine this, intercept tests within the MR Egger and MR Pleiotropy Residual Sum and Outlier (MR-PRESSO) were employed to scrutinize the presence of horizontal pleiotropy in the MR analysis. Furthermore, the MR-PRESSO method was employed to identify and mitigate any potential influence of outliers on the MR analysis results. Employing a leave-one-out strategy, individual SNPs that might unduly impact the MR analysis outcomes were detected. Lastly, the MR Robust Adjusted Profile Score (MR-RAPS) method was invoked to subject the distribution of MR analysis results to a normality test, ensuring robustness in statistical properties.

### Statistical analysis

The data analysis for this study was conducted using R version 4.1.2. The “TwoSampleMR” software package was utilized to execute a two-sample MR analysis, examining the relationship between the exposures (VICNS and IDCNS) and the outcome (KOA). Subsequently, the “MRPRESSO” software package was employed to assess horizontal pleiotropy and identify potential outliers within the MR analysis. The statistical assessment indicated a genetic causality between the exposures (VICNS and IDCNS) and the outcome (KOA) at a significance level of *P* < 0.05. Notably, an OR > 1 signifies a positive genetic causality, while an OR < 1 indicates a negative genetic causality. Furthermore, findings with a *P* > 0.05 suggest a lack of heterogeneity and horizontal pleiotropy within the MR analysis, and aligning with the assumptions of a normal distribution.

## Results

### Genetic causality between VICNS and KOA

When employing a stringent correlation criterion of *P* < 5 × 10^− 8^, no SNPs exhibiting significant correlation were identified for subsequent analysis. Consequently, the correlation criterion was adjusted to *P* < 5 × 10^− 6^. This adjustment revealed a total of 11 SNPs displaying a significant correlation with VICNS (*P* < 5 × 10^− 6^, F statistic > 10), concurrently manifesting within the GWAS summary data of KOA. All 11 identified SNPs satisfied the criterion of MAF > 0.01. Notably, these SNPs exhibited no discernible association with the outcome variable (KOA) or confounding factors such as advanced age and obesity. As a result, these 11 SNPs were selected as IVs for the genetic causal assessment between VICNS and KOA. Noteworthy, our analysis did not identify the presence of palindromic SNPs in the dataset (Supplementary Table 3).

**Fig. 1:**
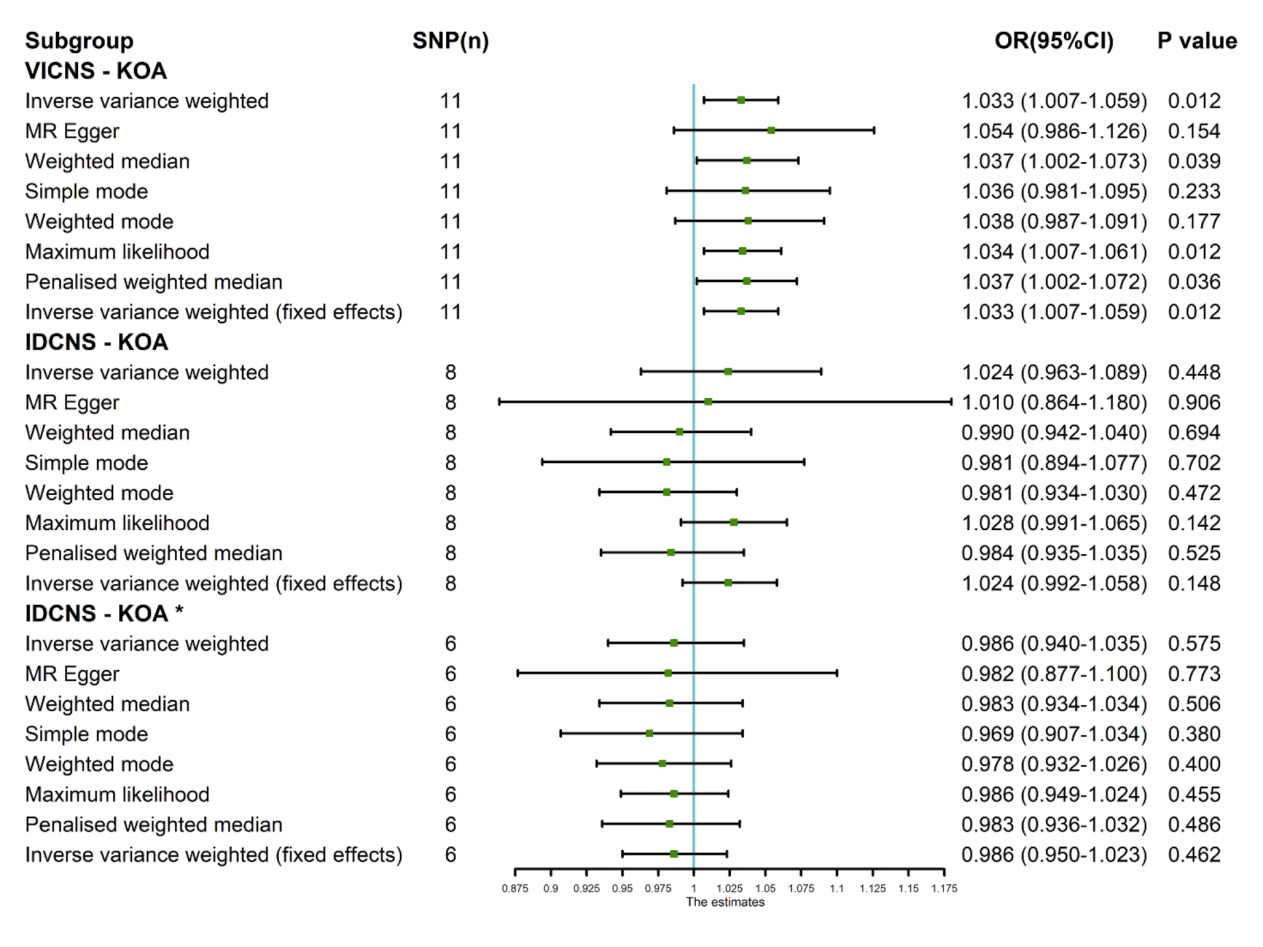
The MR analysis results of exposures (VICNS and IDCNS) and outcome (KOA). The analysis employed eight methods, namely random-effects IVW, MR Egger, weighted median, simple mode, weighted mode, maximum likelihood, penalized weighted median, and fixed-effects IVW

**Fig. 2:**
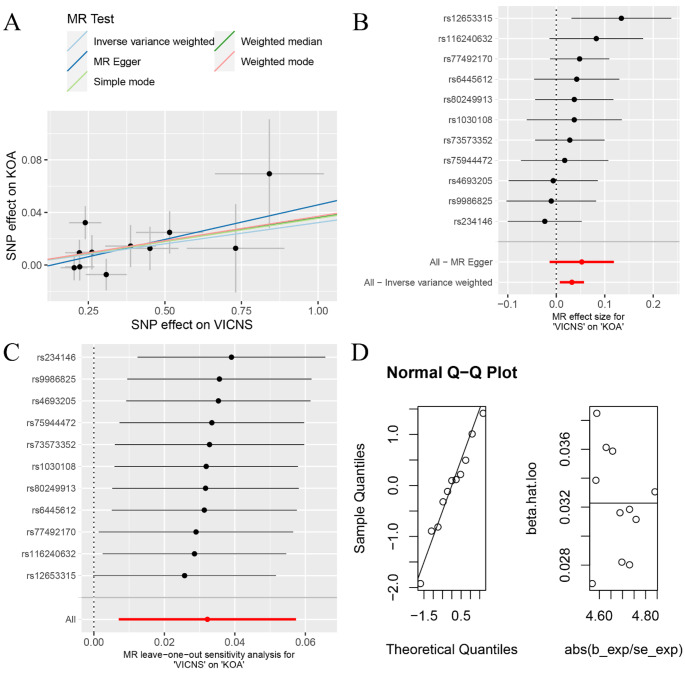
The MR analysis between VICNS and KOA. (**A**) scatter plot; (**B**) forest plot; (**C**) leave-one-out analysis; (**D**) normal distribution

The findings derived from the application of various MR approaches suggest a discernible genetic causal association between VICNS and KOA. Specifically, employing random-effects IVW analysis revealed a suggestive positive relationship (*P* = 0.012, odds ratio [OR] 95% confidence interval [CI] = 1.033 [1.007–1.059]). Consistency in outcomes was observed through the weighted median analysis, further substantiating the suggestive positive relationship identified by random-effects IVW (*P* = 0.039, OR 95% CI = 1.037 [1.002–1.073]). However, alternate methodologies such as MR Egger, simple mode, and weighted mode analyses did not indicate a statistically significant genetic causal link between VICNS and KOA (*P* > 0.05) (Figs. 1 and 2A and B). Furthermore, three validation techniques, namely maximum likelihood estimation, penalised weighted median, and fixed effects IVW, consistently corroborated the presence of a suggestive positive genetic causal relationship between VICNS and KOA (Fig. 1), with statistical significance evident in their respective analyses (maximum likelihood: *P* = 0.012, OR 95% CI = 1.034 [1.007–1.061]; penalised weighted median: *P* = 0.036, OR 95% CI = 1.037 [1.002–1.072]; fixed effects IVW: *P* = 0.012, OR 95% CI = 1.033 [1.007–1.059]).

Cochran’s Q statistic for MR-IVW and Rucker’s Q statistic for MR Egger were utilized, both indicating a lack of heterogeneity (*P* > 0.05). Furthermore, assessments for horizontal pleiotropy through intercept tests in MR Egger and MR-PRESSO revealed no significant evidence of horizontal pleiotropy (*P* > 0.05). MR-PRESSO detection analysis indicated the absence of outliers (Table 1). The robustness of the genetic causality assessment was further confirmed through a leave-one-out analysis, demonstrating that the relationship between VICNS and KOA remained unaffected by the individual SNPs (Fig. 2C). Additionally, MR-RAPS analysis depicted the conformity of the genetic causal assessment between VICNS and KOA with a normal distribution (Table 1; Fig. 2D).

### Genetic causality between IDCNS and KOA

In line with the approach adopted in the VICNS and KOA’s MR analysis, a correlation criterion of *P* < 5 × 10^− 6^ was utilized in our study. We identified a set of eight SNPs that were shared by IDCNS and KOA (*P* < 5 × 10^− 6^, F statistic > 10). These SNPs demonstrated independence from the KOA outcome and potential confounding factors such as advanced age and obesity. Furthermore, these SNPs adhered to the standard MAF > 0.01 and were not characterized as palindromic variants. Subsequently, leveraging these eight SNPs as IVs, we conducted an assessment to ascertain the genetic causality between IDCNS and KOA (Supplementary Table 4).

**Fig. 3:**
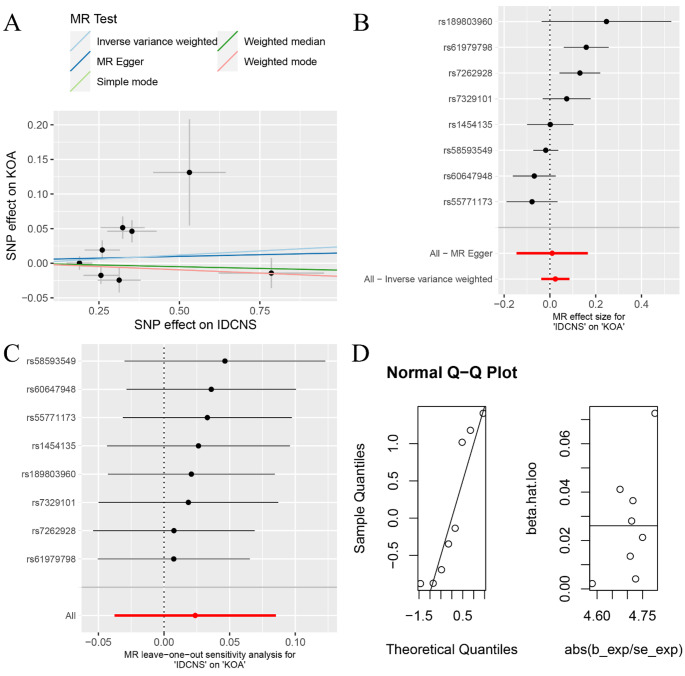
The MR analysis between IDCNS and KOA. (**A**) scatter plot; (**B**) forest plot; (**C**) leave-one-out analysis; (**D**) normal distribution

**Fig. 4:**
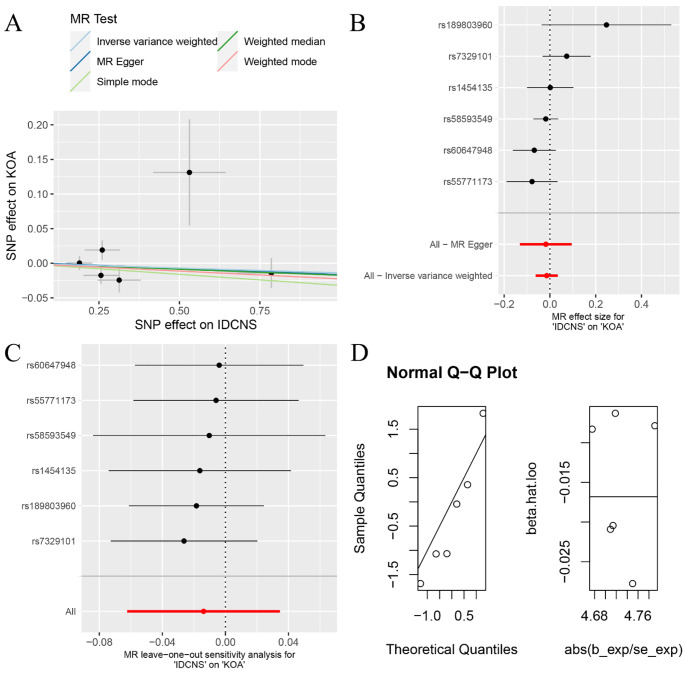
The second round of MR analysis of IDCNS and KOA after deleting two outliers. (**A**) scatter plot; (**B**) forest plot; (**C**) leave-one-out analysis; (**D**) normal distribution

The random-effects IVW analysis indicated an absence of a discernible genetic causal relationship between IDCNS and KOA (*P* = 0.448, OR 95% CI = 1.024 [0.963–1.089]). Consistency across multiple MR methodologies, including MR Egger, weighted median, simple mode, weighted mode, maximum likelihood, penalised weighted median, and fixed effects IVW, mirrored the findings of the random-effects IVW analysis (*P* > 0.05) (Figs. 1 and 3A and B). Notably, the MR-IVW’s Cochran’s Q statistic and MR Egger analysis’ Rucker’s Q statistic revealed significant heterogeneity (*P* < 0.05). While the intercept test in MR Egger’s analysis did not detect horizontal pleiotropy (*P*  >0.05), the MR-PRESSO analysis indicated its presence (*P*  <0.05). And the MR-PRESSO identifying two outliers (rs61979798 and rs7262928) (Table 1). The Leave-one-out analysis effectively discounted the possibility of single-SNP influence on the genetic causal assessment (Fig. 3C). MR-RAPS demonstrated the normal distribution of ​genetic causal assessment outcome (Table 1; Fig. 3D). Subsequent genetic causality assessment, post-elimination of the two outliers, through a second round of analysis, aligning with the initial results, reaffirmed the absence of genetic causation between IDCNS and KOA (*P* = 0.575, OR 95% CI = 0.986 [0.940–1.035]). This finding was supported by seven other MR analysis methods (*P* > 0.05) (Figs. 1 and 4A and B). The second round of MR analysis revealed no heterogeneity, horizontal pleiotropy, or outliers (*P* > 0.05) (Table 1). Importantly, the genetic causal assessment remained unaffected by the influence of a single SNP (Fig. 4C) and followed a normal distribution (Table 1; Fig. 4D).

**Table 1:** Sensitivity analysis of the MR analysis results of exposures and outcomes

Exposure	Outcome	Heterogeneity		Pleiotropy	MR-PRESSO		MR-RAPS
		Cochran’s Q Test (IVW)	Rucker’s Q Test (MR-Egger)	Intercept Test (MR-Egger)	Outliers	Pleiotropy	Normal Distribution
		*P* value	*P* value	*P* value	Number	*P* value	*P* value
VICNS	KOA	0.549	0.495	0.531	0	0.585	0.930
IDCNS	KOA	0.001	0.0003	0.853	2	0.006	0.084
IDCNS	KOA*	0.126	0.072	0.941	0	0.239	–

MR: mendelian randomization; VICNS: viral infections of the central nervous system; IDCNS: inflammatory diseases of the central nervous system; KOA: knee osteoarthritis

## Discussion

This study aims to investigate the causal relationships between the VICNS, the IDCNS, and KOA using MR analysis. The adoption of the MR approach offers a significant methodological advantage by mitigating the influence of confounding factors and reverse causation, thereby enhancing the reliability and validity of the causal inferences drawn. The results indicate a suggestive positive genetic causal association between VICNS and KOA, implying that VICNS may serve as a potential risk factor for the development of KOA. This finding highlights the possibility of a pathophysiological link mediated by neural regulatory mechanisms within the CNS. In contrast, the study did not reveal a significant genetic causal relationship between IDCNS and KOA, although it remains plausible that non-genetic interactions or environmental factors may underlie their potential association. This research contributes to the understanding of the genetic basis of the CNS’s involvement in OA, particularly in the context of KOA, which represents the most prevalent and clinically impactful form of OA. By identifying a genetic connection between VICNS and KOA, this study underscores the importance of further exploring CNS-related mechanisms that may drive OA pathogenesis. Additionally, the absence of a genetic linkage between IDCNS and KOA warrants further investigation into non-genetic factors and the potential interplay of genetic and environmental determinants. This study advances the field by elucidating a partial genetic framework underlying the CNS-OA relationship, it also lays a foundation for future research aimed at uncovering more comprehensive insights into the complex pathophysiology of OA.

Adrenergic receptors (ARs) are pivotal in modulating the sympathetic nervous system (SNS) activation, playing a crucial role in regulating various physiological functions within the body, such as neuronal, cardiovascular, endocrine, inflammatory, and metabolic functions [26, 27]. The acute stress response triggers the release of endogenous catecholamines—norepinephrine (NE) and epinephrine (E)—into the bloodstream, synthesized primarily in the adrenal medulla. NE, primarily derived from peripheral sympathetic nerve endings, alongside E, binds to distinct receptors, inducing varied effects across different tissues [28]. OA is a chronic degenerative joint disorder characterized by articular cartilage degradation, synovitis, osteophyte formation, subchondral osteosclerosis, and ligament and meniscus degeneration [5, 29]. Although NE hasn’t been directly identified within human cartilage, surrounding tissues like synovium and subchondral bone produce and release NE, enabling its diffusion through the cartilage matrix to reach chondrocytes [30]. Moreover, the presence of ARs in human chondrocytes underscores the potential influence of NE secreted by neighboring tissues on cartilage. Extensive research indicates that α2a-AR and β2-AR are the most abundant AR subtypes in human cartilage [31, 32]. Animal studies have demonstrated that α2a-AR activation promotes cartilage degeneration and subchondral bone loss [33]. Similarly, β2-AR-mediated signaling in mesenchymal stem cells leads to subchondral bone loss, with the β2-AR agonist isoproterenol significantly augmenting cartilage degradation [34, 35]. Conversely, conditioned loss of β2-AR attenuated subchondral bone loss, cartilage degradation, and calcification [36]. In vitro analysis of isolated chondrocytes revealed that β2-AR activation stimulated cell growth and inhibited chondrocyte differentiation in mouse growth plate chondrocytes, marked by decreased expression of type II collagen, Indian hedgehog protein (Ihh), and type X collagen through phosphorylation of extracellular signal-regulated kinases1/2 (ERK1/2) and protein kinase A (PKA) [37, 38]. NE also exerts a catabolic role in rat chondrocytes through α2a-AR, reducing aggregate expression and increasing metalloproteinase 3 (MMP3), MMP13, and receptor activator of nuclear factor-KB ligand (RANKL) expression by activating ERK1/2 and PKA [33]. To summarize, both α2a-AR and β2-AR subtypes exacerbate the catabolic effects on articular cartilage degeneration in experimentally induced OA.

The subchondral bone alterations observed in OA encompass several significant changes, including augmented subchondral bone plate thickness, alterations in the trabecular structure of subchondral bone, as well as the development of osteophytes and bone cysts [39]. Early stages of OA exhibit escalated bone resorption, subsequently reflected in elevated levels of urinary N-terminal type I collagen telopeptide (NTX) and C-terminal type I collagen telopeptide (CTX). As OA progresses, there is a notable increase in subchondral bone thickness, evidenced by heightened serum levels of osteocalcin and osteopontin among OA patients [40]. Both bone and bone marrow are highly innervated tissues, with hydroxylase-positive (TH+) fibers establishing direct contact with all bone cells. TH + cells have been identified in the bones of OA patients, with heightened mechanical stress inducing the infiltration of TH + nerve fibers into the subchondral bone [33, 41]. In a rat model inducing unilateral anterior crossbite (UAC), observations revealed the sprouting of TH + nerve fibers and increased subchondral bone NE within the temporomandibular joint, without concurrent alterations in systemic NE concentrations [35]. Moreover, OA induced by UAC in rats showcased an upregulation of β2-AR genes and proteins in subchondral bone mesenchymal stem cells (MSCs) [35]. The use of the β2-AR antagonist propranolol effectively suppressed subchondral bone loss in the temporomandibular joint by diminishing osteoclast activity, while conversely, the β2-AR agonist intensified bone loss through activating osteoclasts. Following UAC induction, increased expression of β2-AR within the temporomandibular joint’s subchondral bone led to PKA activation and elevated expression of RANKL, thereby amplifying osteoclast activity and subsequent subchondral bone loss [35]. Furthermore, investigations have demonstrated the involvement of α2a- and α2c-AR in regulating presynaptic neurotransmitter release. In an α2a/α2c-AR deficient mouse model, the absence of α2a- and α2c-AR resulted in heightened SNS activity, elevated plasma NE levels, decreased bone resorption, and increased bone mass [42].

The initiation of synovial inflammation in OA can be attributed to the introduction of cartilage extracellular matrix fragments. These fragments prompt an inflammatory response by activating resident fibroblasts and macrophages within the synovium. Consequently, these stimulated cells release a cascade of pro-inflammatory cytokines, other mediators of inflammation, deoxyadenosine monophosphates (DAMPs), and enzymes responsible for degrading the extracellular matrix. This sequence of events contributes to the recruitment of immune cells, leading to the thickening of the synovial lining layer and the occurrence of synovial hyperplasia [43]. The continuous release of cytokines and catabolic factors perpetuates the degeneration of adjacent cartilage tissues. Notably, certain pro-inflammatory cytokines such as interleukin-6 (IL-6) and tumor necrosis factor-α (TNF-α) have been identified for their role in inducing peripheral sensitization of joint pain receptors, thus amplifying the perception of pain [6, 44]. In the context of synovial tissue from rats with adjuvant arthritis, NE and E were detected [45]. This finding extends to the identification of TH + sympathetic nerve fibers within the synovium of OA patients, accompanied by the presence of NE. Notably, NE synthesis appears to not only emanate from nerve fibers but also from TH + synovial cells that emerge during increased inflammation in the progression of OA. Moreover, enzymes crucial for NE synthesis, including dopamine decarboxylase (DDC), dopamine-β hydroxylase (DBH), and phenylethanolamine-N-methyltransferase (PNMT), have been observed in synovial cells of OA patients. This further substantiates the capacity of OA synovial cells to produce the neurotransmitter NE [46]. Studies involving mixed synovial cells isolated from OA and rheumatoid arthritis (RA) patients demonstrated heightened levels of cytoplasmic catecholamines and the β2 agonist formoterol, activating PKA via β2-AR. This activation subsequently elevates the levels of cyclic adenosine monophosphate (cAMP) and cyclic AMP-response element-binding (CREB) proteins, leading to the downregulation of TNF-α release [46]. Furthermore, NE has been reported to inhibit the secretion of TNF-α and IL-8 mediated by β2-AR in synovial macrophages of patients with OA and RA in subsequent studies [47]. These findings collectively underscore the intricate involvement of NE in modulating inflammatory pathways within the synovial environment, offering potential insights into therapeutic strategies for managing OA-associated inflammation.

Interferons (IFNs) exert their biological effects by binding to specific receptors on the cell surface, initiating intracellular signaling cascades that drive the transcription of interferon-stimulated genes (ISGs). ISGs, and the proteins they encode, are pivotal in mediating the diverse functions of IFNs, including robust antiviral defenses and immunomodulation [48]. Notably, ISGs operate in a cell- and region-specific manner within the CNS to inhibit viral invasion and replication. This specificity is essential for mitigating VICNS, a region with limited regenerative capacity and high sensitivity to inflammation-induced damage. Upon viral infection, IFN production is rapidly induced in peripheral tissues, serving as an early line of defense against viral pathogenicity [49]. The peripherally initiated IFN response can stimulate ISG expression in the brain, highlighting the interconnectedness of systemic and CNS immune responses. For example, peripheral IFN-α can traverse the blood-brain barrier and directly activate IFN-α/β receptor (IFNAR) signaling in microglial cells, resulting in the upregulation of a suite of ISGs. This early induction of ISG expression within the CNS is critical in controlling viral replication and protecting neural tissues from extensive damage [50]. Beyond their established antiviral and immunoregulatory roles, IFNs are implicated in a broad range of pathological and physiological processes. They are central to the progression of allergic reactions, chronic inflammatory diseases, autoimmune disorders, transplant rejection, and certain viral infections [51]. Moreover, IFNs are increasingly recognized as integral components of neuroinflammatory networks, underscoring their complex role in CNS homeostasis and disease [48]. A key mediator within the IFN signaling cascade is interferon regulatory factor 1 (IRF1), a transcription factor traditionally associated with immune regulation. Recent insights suggest that IRF1 extends its influence beyond immunomodulation to DNA repair. Molecular analyses of IRF1 DNA binding sites reveal its involvement in DNA damage surveillance, particularly in chondrocytes of OA. This function is vital for mitigating oxidative stress, a known risk factor for OA. IRF1 appears to exert a unique chondroprotective role independent of its classical immune-related activities. In articular cartilage, IRF1 ensures genomic integrity by surveilling and repairing DNA damage incurred during mechanical stress events, such as joint loading. The absence or reduced activity of IRF1 compromises this surveillance mechanism, leading to an accumulation of senescent chondrocytes. This senescence, in turn, increases vulnerability to cellular dysfunction and contributes significantly to the progression of OA. Such findings underscore the multifaceted role of IRF1 in both maintaining cartilage health and modulating the disease trajectory of OA [52]. The immunological complexity of OA is further highlighted by the role of IRF5, another transcriptional regulator. Elevated levels of IRF5 expression have been detected in circulating monocytes of OA patients, mediated by synovial fluid signaling. As a downstream target of p53, IRF5 is upregulated during DNA damage and can also be induced through Toll-like receptor signaling pathways [53]. This dual regulatory mechanism underscores its significance in the inflammatory microenvironment of OA. In the context of viral infections, delayed activation of type I IFN signaling pathways in OA patients exacerbates their vulnerability to pathogens like the Ross River virus. This virus not only promotes the expression of osteoclastic factors but also shifts the RANKL/OPG ratio in favor of bone resorption, accelerating bone remodeling and potentially worsening OA-associated joint degeneration. These findings highlight the interplay between viral infections, IFN pathways, and the molecular mechanisms driving OA progression [54]. The intricate roles of IFNs, ISGs, and associated regulatory factors like IRF1 and IRF5 illuminate a complex network of protective and pathological processes within the CNS and OA articular tissues. While these molecules offer potent antiviral defenses and immunoregulatory capacities, their dysregulation in specific contexts, such as OA, underscores their dual-edged nature. Future studies aimed at modulating IFN signaling pathways could unlock therapeutic strategies to mitigate disease progression while preserving the beneficial aspects of these critical molecular mediators.

Programmed cell death (PCD) represents a vital biological mechanism through which cells respond to infection and injury. During viral infections, the primary function of PCD is believed to involve constraining viral replication and dissemination by depriving the pathogen of cellular resources needed for propagation. PCD manifests in various forms, broadly categorized as non-lytic (e.g., apoptosis) or lytic (e.g., necrosis and pyroptosis). Non-lytic cell death is generally associated with low inflammatory potential, whereas lytic cell death triggers robust inflammatory responses. This dichotomy highlights the nuanced regulation of PCD, especially within the CNS, where dysregulated cell death can lead to deleterious neuroinflammation and tissue pathology [55]. Apoptosis, a well-characterized form of PCD, operates through two primary pathways: intrinsic and extrinsic. Both pathways are actively implicated in VICNS. The intrinsic pathway is stimulated by disruptions in cellular homeostasis, which are commonly observed during infections. These disruptions include DNA damage, oxidative stress mediated by reactive oxygen species (ROS), and endoplasmic reticulum stress. Conversely, the extrinsic pathway is initiated by external cues, such as the activation of “death receptors” or the lack of stimulation from “dependence receptors,” which can induce apoptosis in ligand-depleted conditions [55]. Activated CD8 T cells serve as a primary source of extrinsic apoptotic signals during viral infections. These cells often express Fas ligand (FASL), a member of the TNF cytokine family, which binds to Fas receptors on target cells to induce apoptosis. However, within the CNS, the apoptosis elicited by such mechanisms often exacerbates disease through direct and indirect pro-inflammatory effects. Specifically, apoptosis within the CNS has been linked to neuroinflammation and subsequent tissue damage, which can override the initial protective intent of cell death [56]. Interestingly, phagocytes that clear apoptotic cells can modulate inflammation by secreting anti-inflammatory cytokines such as IL-10. IL-10’s immunomodulatory effects are particularly relevant during CNS infections, where it plays a pivotal role in regulating glial cell activation and mitigating excessive neuroinflammatory responses [57, 58]. Indeed, multiple studies underscore IL-10’s importance in maintaining immune homeostasis during neuroinflammation [59, 60]. The role of PCD extends beyond the CNS, influencing the pathophysiology of conditions like OA. OA is characterized by pathological mechanisms including cartilage degradation, subchondral bone remodeling, and synovial inflammation, which culminate in joint space narrowing, osteophyte formation, and progressive tissue destruction [61, 62]. Among these processes, cartilage degradation is a defining hallmark. Chondrocytes, the primary cellular component of cartilage, play a crucial role in maintaining cartilage homeostasis by regulating the turnover of the extracellular matrix. This balance relies on the tightly controlled processes of chondrocyte proliferation, differentiation, and apoptosis [63]. Apoptosis is prominent in OA cartilage, with the proportion of apoptotic chondrocytes varying between less than 1% and approximately 20%, depending on the stage and severity of the disease [64]. Importantly, the degree of chondrocyte apoptosis correlates positively with the extent of cartilage degeneration, further emphasizing its central role in OA progression [56]. Inflammatory mediators such as ROS, nitric oxide (NO), IL-1β, TNF-α, and Fas ligand contribute to the induction of chondrocyte apoptosis in OA. Several signaling pathways are implicated in OA pathogenesis, including the NF-κB pathway, Wnt signaling, and the Notch pathway. These pathways exhibit complex, often biphasic roles in chondrocyte fate determination. For instance, depending on contextual factors, these pathways may either promote or suppress chondrocyte apoptosis and extracellular matrix degradation. Dysregulated activation of these pathways contributes to the progressive nature of OA by exacerbating inflammatory and catabolic processes [64]. Programmed cell death, while essential for maintaining cellular homeostasis, plays complex and context-dependent roles in disease settings. In the CNS, apoptotic mechanisms may shift from protective to pathological, aggravating neuroinflammation and tissue damage. Similarly, in OA, the misregulation of chondrocyte apoptosis and associated signaling pathways underscores their contributions to disease severity. Further elucidation of these pathways could open avenues for targeted therapeutic interventions aimed at modulating PCD to preserve tissue integrity and mitigate disease progression.

The role of chemokines in mediating the immune response to viral infections in the CNS is largely centered on their ability to recruit and activate antigen-specific lymphocytes. Early after viral infection, chemokines are rapidly expressed and act as key regulators of the innate immune response, serving to coordinate cellular defenses against invading pathogens. For example, the chemokine CXCL10 plays a crucial role in the CNS defense against coronavirus infections. Its expression amplifies innate immune responses, curtails disease progression, and significantly improves survival rates. This protective effect is predominantly attributed to the enhanced recruitment and activation of natural killer (NK) cells within the CNS. These NK cells facilitate viral clearance by reducing viral titers through mechanisms dependent on interferon-gamma (IFN-γ) secretion, thereby emphasizing the importance of CXCL10 in antiviral immunity [65]. Beyond its interaction with NK cells, chemokine ligand 3 (CCL3) is instrumental in shaping the host’s adaptive immune response following VICNS. During mouse hepatitis virus (MHV) infection, CCL3 is essential for the activation and function of dendritic cells (DCs), particularly the CD11c + CD11b + CD8α- subset. CCL3 facilitates the maturation, activation, and migration of these DCs to cervical lymph nodes, enhancing antigen presentation and T-cell priming. Impaired activation of CD8α- DCs has been shown to reduce IFN-γ expression while increasing IL-10 production by virus-specific T cells. This suggests that CCL3’s influence on DCs enhances their capacity to activate T cells, leading to a more robust and effective host response to viral infections. These findings highlight the interplay between chemokines and antigen-presenting cells in shaping antiviral immunity within the CNS [66]. In OA, chemokines have similarly been implicated as mediators of both tissue repair and disease progression. Synovial fluid, which is enriched with chemokines, has been shown to stimulate the migration of subchondral progenitor cells. Among these chemokines, CXCL10 stands out due to its interaction with CXCR3, the chemokine receptor expressed at the highest levels on subchondral progenitor cells. This CXCL10-CXCR3 axis is believed to facilitate the recruitment of mesenchymal progenitor cells to areas of microfracture in subchondral bone, potentially contributing to tissue repair. However, the same chemokine signaling can also drive pathological processes in OA by recruiting immune cells to the synovium [67]. During OA progression, a variety of immune cell populations are involved in processes such as cartilage damage, bone erosion, and bone resorption. NK cells and neutrophils have been identified as key contributors to OA pathogenesis, with studies showing that they are among the first immune cells to infiltrate the synovium. These cells exert pro-inflammatory and destructive effects that exacerbate joint damage. The CXCL10-CXCR3 axis plays a central role in this process, driving the recruitment and activation of NK cells and neutrophils within OA-affected synovial tissue. Elevated CXCL10 expression in the synovium further correlates with disease severity, reinforcing its pathogenic significance. Interactions between these immune cells, mediated through CXCL10-CXCR3 signaling, create a feedback loop that amplifies local inflammation and tissue degradation, ultimately accelerating OA progression [68]. Chemokines are pivotal regulators of immune responses in both VICNS and OA pathogenesis, with their roles spanning from mobilizing innate immune defenses to modulating adaptive responses. CXCL10 emerges as a critical chemokine in both contexts, demonstrating versatility in orchestrating protective and pathogenic immune processes. In viral infections, CXCL10 supports effective antiviral defenses, while in OA, its aberrant signaling contributes to chronic inflammation and tissue destruction. Understanding the dualistic roles of chemokines offers opportunities to develop targeted therapies that either enhance their protective functions or mitigate their contribution to disease progression. Further research into chemokine signaling pathways may uncover novel interventions for a wide range of inflammatory and degenerative disorders.

The CNS plays an integral role in the regulation of skeletal system homeostasis, reflecting its broad physiological and pathological influence [9]. Anatomically, sensory nerves within the skeletal system establish interconnections with the hypothalamus, particularly the ventromedial nucleus (VMH). The VMH is a critical hub for autonomic nervous system (ANS) modulation and regulates diverse biological processes, including weight management, glucose homeostasis, emotional responses, and reproductive functions [69]. Such connectivity highlights the interplay between the CNS and the skeletal framework in both normal and pathological states. The CNS is a central regulator of the pathological transformations associated with OA, including those affecting cartilage integrity, subchondral bone remodeling, and synovial membrane inflammation. Beyond regulating these localized pathological mechanisms, recent studies point to a potential genetic link between CNS functions and OA pathology. Although our research did not identify a definitive genetic causative relationship between the IDCNS and KOA, findings suggest a positive genetic causal association between VICNS and KOA development. These results underscore the possibility that VICNS contributes to the initiation or progression of KOA. Given the chronic and progressive nature of OA, acute perturbations in VICNS are unlikely to be sufficient to directly trigger KOA. Instead, chronic disruptions in CNS function may act as risk factors that predispose individuals to KOA. This perspective aligns with emerging evidence that chronic neural and inflammatory signaling alterations within the CNS can exacerbate peripheral joint degeneration. The findings emphasize the need for further exploration into how chronic CNS dysfunction and genetic predisposition intertwine to modulate KOA susceptibility and progression. The potential for VICNS to contribute to KOA highlights a novel dimension of OA research that bridges neurobiology with joint pathophysiology. Future studies should aim to identify specific genetic and neural pathways that mediate this interaction and determine how these pathways are modulated. Such insights could open new therapeutic avenues targeting CNS regulatory mechanisms to mitigate KOA risk or slow disease progression. By integrating CNS dysfunction into the broader context of OA etiology, a more comprehensive understanding of the disease may emerge, fostering the development of multifaceted treatment approaches.

This study has certain limitations that merit careful consideration. First and foremost, the investigation predominantly centers on the European population, which inherently restricts the generalizability of the findings to diverse ethnic and demographic groups. This demographic specificity underscores the need for subsequent studies encompassing more heterogeneous populations to validate and expand upon the present results. Secondly, due to constraints within the available GWAS dataset, it was not possible to differentiate VICNS into acute and chronic categories. This limitation precluded a more nuanced stratification and analysis of the variable, potentially overlooking differential genetic or pathophysiological pathways underlying these distinct forms of CNS influence. Future research with datasets offering finer granularity is essential to address this gap. Thirdly, while this study specifically focuses on KOA, it should be noted that OA encompasses a range of joint-specific manifestations. Although KOA is widely recognized as the most clinically prevalent and burdensome form of OA, the exclusive focus on KOA does not fully encapsulate the broader complexity and variability of OA phenotypes. This highlights the importance of extending future investigations to other joint sites affected by OA for a more comprehensive understanding of the disease. In summary, these limitations highlight areas for improvement in future research to enhance the robustness, applicability, and scope of the findings. Despite these constraints, the study contributes valuable insights into the genetic underpinnings of KOA, reaffirming its clinical importance and setting the stage for more targeted and inclusive investigations in the field.

## Conclusion

The findings of the current study highlight a suggestive positive genetic causal association between VICNS and KOA, thereby implying a potential influence of CNS pathways on the development of KOA. In contrast, no significant genetic causal relationship was observed between IDCNS and KOA, suggesting the presence of pathway-specific mechanisms within the CNS that may selectively impact OA processes. These results provide robust genetic evidence supporting the involvement of CNS mechanisms in the regulation of OA pathophysiology. The identification of a CNS-OA axis enriches our understanding of OA as a complex, multi-system disease rather than a localized joint disorder. Importantly, this perspective underscores the critical role of neural regulatory pathways in shaping the onset and progression of OA. From a clinical standpoint, these findings advocate for a paradigm shift in OA management. Therapeutic strategies should not solely focus on the biomechanical and inflammatory dimensions of OA but also consider the central regulatory mechanisms within the CNS. Such a holistic approach could pave the way for novel interventions targeting CNS pathways, ultimately contributing to improved disease management and patient outcomes.

## Electronic supplementary material

Below is the link to the electronic supplementary material.

Supplementary Material 1

## Data Availability

This study utilized publicly available datasets, which were obtained from the FinnGen consortium (https://www.finngen.fi/) and IEU OpenGWAS database (https://gwas.mrcieu.ac.uk/)..
