# Supplementary material for: Viral infections of the central nervous system increase the risk of knee osteoarthritis: a two-sample mendelian randomization study
Source: Aging Clin Exp Res. 2025 Jan 21;37(1):30. doi: 10.1007/s40520-025-02927-7 (PMC11750930; doi:10.1007/s40520-025-02927-7)
Supplement: Supplementary file 1 — Supplementary Material 1 [file 40520_2025_2927_MOESM1_ESM.doc]

**Supplementary Table 1:** STROBE-MR checklist of MR study.

| **Item No** | **Section** | **Checklist item** |
| --- | --- | --- |
| 1 | **Title** | Viral infections of the central nervous system increase the risk of knee osteoarthritis: a two-sample mendelian randomization study |
| 2 | **Abstract** | Osteoarthritis (OA) represents a condition under the influence of central nervous system (CNS) regulatory mechanisms. This investigation aims to examine the causal association between viral infections of the central nervous system (VICNS) and inflammatory diseases of the central nervous system (IDCNS) and knee osteoarthritis (KOA) at the genetic level. In this investigation, VICNS and IDCNS were considered as primary exposure variables, while KOA served as the primary outcome. Employing a two-sample mendelian randomization (MR) approach, we conducted an analysis utilizing summary data derived from genome-wide association studies (GWAS). The result of this study has elucidated a suggestive positive genetic causal link between the VICNS and KOA. However, no such genetic causal relationship was observed between the IDCNS and KOA. These findings substantiate the genetic underpinnings supporting the association between the CNS and OA. |
|  | **Introduction** |  |
| 3 | Background | Osteoarthritis (OA) represents the predominant chronic joint ailment and is recognized as an age-related degenerative disease. Patients diagnosed with OA are predisposed to the dysregulation of various central feedback mechanisms governing sympathetic tone, inflammation, circadian rhythms encompassing both central and peripheral clocks, gut microbiota, metabolic redox, and the overall pathology of affected joints. Notably, recent attention has been directed toward understanding the intricate association between OA and the central nervous system (CNS). Investigations have indicated that the experience of OA-related pain is, in part, attributed to CNS processes, with identified irregularities in the processing of nociceptive stimuli within the CNS that bear resemblance to those observed in other chronic pain conditions. Current research underscores the crucial role of the nervous system in regulating skeletal metabolism, highlighting the significance of skeletal interoception in maintaining bone homeostasis. The CNS emerges as a pivotal regulator of the skeletal system, exhibiting robust sensory and sympathetic innervation vital for bone homeostasis and pain modulation. Bones are extensively innervated sensorily, and the interaction between the CNS and bone physiology has been elucidated through viral tracer experiments and immunofluorescence, elucidating the pathway from bone sensory neurons to the central nervous system. It is increasingly evident that OA transcends local joint affliction, with the CNS exerting a significant influence on its progression. Consequently, exploring the regulatory impact of the CNS on OA represents a promising avenue for research to mitigate disease progression. Therefore, further investigation into the multifaceted relationship between the CNS and OA across various levels is imperative to inform clinical decision-making in managing OA. |
| 4 | Objectives | OA represents a condition under the influence of CNS regulatory mechanisms. This investigation aims to examine the causal association between VICNS and IDCNS and KOA at the genetic level. |
|  | **Methods** |  |
| 5 | Study design | In this investigation examining the genetic causal relationship between VICNS and IDCNS as exposures and KOA as outcomes, a two-sample MR analysis was conducted. The analysis adhered rigorously to the fundamental assumptions of MR methodology. |
| 6 | Assumptions |  |
|  | a) | The IVs exhibited a robust association with the exposures (VICNS and IDCNS) |
|  | b) | The IVs remained independent of both the outcome (KOA) and potential confounding variables. |
|  | c) | The effects of the IVs on the outcomes were solely mediated through the exposures. |
| 7 | Data sources |  |
|  | a) | We acquired the GWAS summary data for two distinct exposures, VICNS and IDCNS, from the Finnish consortium, accessible at https://www.finngen.fi/. |
|  | b) | Additionally, the IEU OpenGWAS database (https://gwas.mrcieu.ac.uk/) contains GWAS summary data concerning the outcome known as KOA. |
| 8 | MR analysis |  |
|  | a) | Main method: random-effects IVW. |
|  | b) | Supplementary methods: MR Egger, Weighted median, Simple mode, and Weighted mode. |
|  | c) | Verification methods: Maximum likelihood, Penalised weighted median, and IVW (fixed effects). |
| 9 | Sensitivity analysis |  |
|  | a) | Heterogeneity: Cochran’s Q statistic for MR-IVW analyses and Rucker’s Q statistic for MR Egger analyses. |
|  | b) | Horizontal pleiotropy: intercept test of MR Egger and MR-PRESSO. |
|  | c) | Outliers: MR-PRESSO. |
|  | d) | Leave one out: detect whether the causal relationship was influenced by a single SNP. |
|  | e) | Normal distribution: MR-RAPS. |
| 10 | Software | R software (version 4.1.2) |
|  | **Results** |  |
| 11 | Descriptive data |  |
|  | a) | VICNS and KOA: 11 SNPs were used as IVs. |
|  | b) | IDCNS and KOA:8 SNPs were used as IVs. |
| 12 | Main results |  |
|  | a) | VICNS and KOA: positive genetic causal relationship. |
|  | b) | IDCNS and KOA: no genetic causal relationship. |
| 13 | Sensitivity analysis |  |
|  | a) | No heterogeneity. |
|  | b) | No horizontal pleiotropy. |
|  | c) | No outliers. |
|  | d) | The "leave one out" analysis indicated that the MR analysis results were not driven by a single SNP. |
|  | e | The MR analysis followed a normal distribution. |
|  | **Discussion** |  |
| 14 | Key results | The present study endeavors to scrutinize the causative interplay between the VICNS and the IDCNS and KOA utilizing MR analysis. This analytical approach is impervious to confounding variables and reverse causation, ensuring a robust investigation. The outcomes unveiled a suggestive positive genetic causal nexus between VICNS and KOA, suggest that VICNS may be a risk factor for the occurrence of KOA. Conversely, the findings of this research did not substantiate a genetic causal association between IDCNS and KOA, although non-genetic links cannot be discounted. This inquiry contributes, to a certain extent, to elucidating the genetic underpinnings of the relationship between the CNS and OA. |
| 15 | Interpretation |  |
|  | a) | Adrenergic receptors (ARs) are pivotal in modulating the sympathetic nervous system (SNS) activation, playing a crucial role in regulating various physiological functions within the body, such as neuronal, cardiovascular, endocrine, inflammatory, and metabolic functions. The acute stress response triggers the release of endogenous catecholamines—norepinephrine (NE) and epinephrine (E)—into the bloodstream, synthesized primarily in the adrenal medulla. OA is a chronic degenerative joint disorder characterized by articular cartilage degradation, synovitis, osteophyte formation, subchondral osteosclerosis, and ligament and meniscus degeneration. Although NE hasn't been directly identified within human cartilage, surrounding tissues like synovium and subchondral bone produce and release NE, enabling its diffusion through the cartilage matrix to reach chondrocytes. Moreover, the presence of ARs in human chondrocytes underscores the potential influence of NE secreted by neighboring tissues on cartilage. Extensive research indicates that α2a-AR and β2-AR are the most abundant AR subtypes in human cartilage. Animal studies have demonstrated that α2a-AR activation promotes cartilage degeneration and subchondral bone loss. Similarly, β2-AR-mediated signaling in mesenchymal stem cells leads to subchondral bone loss, with the β2-AR agonist isoproterenol significantly augmenting cartilage degradation. Conversely, conditioned loss of β2-AR attenuated subchondral bone loss, cartilage degradation, and calcification. In vitro analysis of isolated chondrocytes revealed that β2-AR activation stimulated cell growth and inhibited chondrocyte differentiation in mouse growth plate chondrocytes, marked by decreased expression of type II collagen, Indian hedgehog protein (Ihh), and type X collagen through phosphorylation of extracellular signal-regulated kinases1/2 (ERK1/2) and protein kinase A (PKA). NE also exerts a catabolic role in rat chondrocytes through α2a-AR, reducing aggregate expression and increasing metalloproteinase 3 (MMP3), MMP13, and receptor activator of nuclear factor-KB ligand (RANKL) expression by activating ERK1/2 and PKA. To summarize, both α2a-AR and β2-AR subtypes exacerbate the catabolic effects on articular cartilage degeneration in experimentally induced OA. |
|  | b) | The subchondral bone alterations observed in OA encompass several significant changes, including augmented subchondral bone plate thickness, alterations in the trabecular structure of subchondral bone, as well as the development of osteophytes and bone cysts. Early stages of OA exhibit escalated bone resorption, subsequently reflected in elevated levels of urinary N-terminal type I collagen telopeptide (NTX) and C-terminal type I collagen telopeptide (CTX). As OA progresses, there is a notable increase in subchondral bone thickness, evidenced by heightened serum levels of osteocalcin and osteopontin among OA patients. Both bone and bone marrow are highly innervated tissues, with hydroxylase-positive (TH+) fibers establishing direct contact with all bone cells. TH+ cells have been identified in the bones of OA patients, with heightened mechanical stress inducing the infiltration of TH+ nerve fibers into the subchondral bone. In a rat model inducing unilateral anterior crossbite (UAC), observations revealed the sprouting of TH+ nerve fibers and increased subchondral bone NE within the temporomandibular joint, without concurrent alterations in systemic NE concentrations. Moreover, OA induced by UAC in rats showcased an upregulation of β2-AR genes and proteins in subchondral bone mesenchymal stem cells (MSCs). The use of the β2-AR antagonist propranolol effectively suppressed subchondral bone loss in the temporomandibular joint by diminishing osteoclast activity, while conversely, the β2-AR agonist intensified bone loss through activating osteoclasts. Following UAC induction, increased expression of β2-AR within the temporomandibular joint's subchondral bone led to PKA activation and elevated expression of RANKL, thereby amplifying osteoclast activity and subsequent subchondral bone loss. Furthermore, investigations have demonstrated the involvement of α2a- and α2c-AR in regulating presynaptic neurotransmitter release. In an α2a/α2c-AR deficient mouse model, the absence of α2a- and α2c-AR resulted in heightened SNS activity, elevated plasma NE levels, decreased bone resorption, and increased bone mass. |
|  | c) | The initiation of synovial inflammation in OA can be attributed to the introduction of cartilage extracellular matrix fragments. These fragments prompt an inflammatory response by activating resident fibroblasts and macrophages within the synovium. Consequently, these stimulated cells release a cascade of pro-inflammatory cytokines, other mediators of inflammation, deoxyadenosine monophosphates (DAMPs), and enzymes responsible for degrading the extracellular matrix. This sequence of events contributes to the recruitment of immune cells, leading to the thickening of the synovial lining layer and the occurrence of synovial hyperplasia. The continuous release of cytokines and catabolic factors perpetuates the degeneration of adjacent cartilage tissues. Notably, certain pro-inflammatory cytokines such as interleukin-6 (IL-6) and tumor necrosis factor-α (TNF-α) have been identified for their role in inducing peripheral sensitization of joint pain receptors, thus amplifying the perception of pain. In the context of synovial tissue from rats with adjuvant arthritis, NE and E were detected. This finding extends to the identification of TH+ sympathetic nerve fibers within the synovium of OA patients, accompanied by the presence of NE. Notably, NE synthesis appears to not only emanate from nerve fibers but also from TH+ synovial cells that emerge during increased inflammation in the progression of OA. Moreover, enzymes crucial for NE synthesis, including dopamine decarboxylase (DDC), dopamine-β hydroxylase (DBH), and phenylethanolamine-N-methyltransferase (PNMT), have been observed in synovial cells of OA patients. This further substantiates the capacity of OA synovial cells to produce the neurotransmitter NE. Studies involving mixed synovial cells isolated from OA and rheumatoid arthritis (RA) patients demonstrated heightened levels of cytoplasmic catecholamines and the β2 agonist formoterol, activating PKA via β2-AR. This activation subsequently elevates the levels of cyclic adenosine monophosphate (cAMP) and cyclic AMP-response element-binding (CREB) proteins, leading to the downregulation of TNF-α release. Furthermore, NE has been reported to inhibit the secretion of TNF-α and IL-8 mediated by β2-AR in synovial macrophages of patients with OA and RA in subsequent studies. These findings collectively underscore the intricate involvement of NE in modulating inflammatory pathways within the synovial environment, offering potential insights into therapeutic strategies for managing OA-associated inflammation. |
|  | d) | Interferons (IFNs) exert their biological effects by binding to specific receptors on the cell surface, initiating intracellular signaling cascades that drive the transcription of interferon-stimulated genes (ISGs). ISGs, and the proteins they encode, are pivotal in mediating the diverse functions of IFNs, including robust antiviral defenses and immunomodulation. Notably, ISGs operate in a cell- and region-specific manner within the CNS to inhibit viral invasion and replication. This specificity is essential for mitigating VICNS, a region with limited regenerative capacity and high sensitivity to inflammation-induced damage. Upon viral infection, IFN production is rapidly induced in peripheral tissues, serving as an early line of defense against viral pathogenicity. The peripherally initiated IFN response can stimulate ISG expression in the brain, highlighting the interconnectedness of systemic and CNS immune responses. For example, peripheral IFN-α can traverse the blood-brain barrier and directly activate IFN-α/β receptor (IFNAR) signaling in microglial cells, resulting in the upregulation of a suite of ISGs. This early induction of ISG expression within the CNS is critical in controlling viral replication and protecting neural tissues from extensive damage. Beyond their established antiviral and immunoregulatory roles, IFNs are implicated in a broad range of pathological and physiological processes. They are central to the progression of allergic reactions, chronic inflammatory diseases, autoimmune disorders, transplant rejection, and certain viral infections. Moreover, IFNs are increasingly recognized as integral components of neuroinflammatory networks, underscoring their complex role in CNS homeostasis and disease. A key mediator within the IFN signaling cascade is interferon regulatory factor 1 (IRF1), a transcription factor traditionally associated with immune regulation. Recent insights suggest that IRF1 extends its influence beyond immunomodulation to DNA repair. Molecular analyses of IRF1 DNA binding sites reveal its involvement in DNA damage surveillance, particularly in chondrocytes of OA. This function is vital for mitigating oxidative stress, a known risk factor for OA. IRF1 appears to exert a unique chondroprotective role independent of its classical immune-related activities. In articular cartilage, IRF1 ensures genomic integrity by surveilling and repairing DNA damage incurred during mechanical stress events, such as joint loading. The absence or reduced activity of IRF1 compromises this surveillance mechanism, leading to an accumulation of senescent chondrocytes. This senescence, in turn, increases vulnerability to cellular dysfunction and contributes significantly to the progression of OA. Such findings underscore the multifaceted role of IRF1 in both maintaining cartilage health and modulating the disease trajectory of OA. The immunological complexity of OA is further highlighted by the role of IRF5, another transcriptional regulator. Elevated levels of IRF5 expression have been detected in circulating monocytes of OA patients, mediated by synovial fluid signaling. As a downstream target of p53, IRF5 is upregulated during DNA damage and can also be induced through Toll-like receptor signaling pathways. This dual regulatory mechanism underscores its significance in the inflammatory microenvironment of OA. In the context of viral infections, delayed activation of type I IFN signaling pathways in OA patients exacerbates their vulnerability to pathogens like the Ross River virus. This virus not only promotes the expression of osteoclastic factors but also shifts the RANKL/OPG ratio in favor of bone resorption, accelerating bone remodeling and potentially worsening OA-associated joint degeneration. These findings highlight the interplay between viral infections, IFN pathways, and the molecular mechanisms driving OA progression. The intricate roles of IFNs, ISGs, and associated regulatory factors like IRF1 and IRF5 illuminate a complex network of protective and pathological processes within the CNS and OA articular tissues. While these molecules offer potent antiviral defenses and immunoregulatory capacities, their dysregulation in specific contexts, such as OA, underscores their dual-edged nature. Future studies aimed at modulating IFN signaling pathways could unlock therapeutic strategies to mitigate disease progression while preserving the beneficial aspects of these critical molecular mediators. |
|  | e) | Programmed cell death (PCD) represents a vital biological mechanism through which cells respond to infection and injury. During viral infections, the primary function of PCD is believed to involve constraining viral replication and dissemination by depriving the pathogen of cellular resources needed for propagation. PCD manifests in various forms, broadly categorized as non-lytic (e.g., apoptosis) or lytic (e.g., necrosis and pyroptosis). Non-lytic cell death is generally associated with low inflammatory potential, whereas lytic cell death triggers robust inflammatory responses. This dichotomy highlights the nuanced regulation of PCD, especially within the CNS, where dysregulated cell death can lead to deleterious neuroinflammation and tissue pathology. Apoptosis, a well-characterized form of PCD, operates through two primary pathways: intrinsic and extrinsic. Both pathways are actively implicated in VICNS. The intrinsic pathway is stimulated by disruptions in cellular homeostasis, which are commonly observed during infections. These disruptions include DNA damage, oxidative stress mediated by reactive oxygen species (ROS), and endoplasmic reticulum stress. Conversely, the extrinsic pathway is initiated by external cues, such as the activation of "death receptors" or the lack of stimulation from "dependence receptors," which can induce apoptosis in ligand-depleted conditions. Activated CD8 T cells serve as a primary source of extrinsic apoptotic signals during viral infections. These cells often express Fas ligand (FASL), a member of the TNF cytokine family, which binds to Fas receptors on target cells to induce apoptosis. However, within the CNS, the apoptosis elicited by such mechanisms often exacerbates disease through direct and indirect pro-inflammatory effects. Specifically, apoptosis within the CNS has been linked to neuroinflammation and subsequent tissue damage, which can override the initial protective intent of cell death. Interestingly, phagocytes that clear apoptotic cells can modulate inflammation by secreting anti-inflammatory cytokines such as IL-10. IL-10's immunomodulatory effects are particularly relevant during CNS infections, where it plays a pivotal role in regulating glial cell activation and mitigating excessive neuroinflammatory responses. Indeed, multiple studies underscore IL-10's importance in maintaining immune homeostasis during neuroinflammation. The role of PCD extends beyond the CNS, influencing the pathophysiology of conditions like OA. OA is characterized by pathological mechanisms including cartilage degradation, subchondral bone remodeling, and synovial inflammation, which culminate in joint space narrowing, osteophyte formation, and progressive tissue destruction. Among these processes, cartilage degradation is a defining hallmark. Chondrocytes, the primary cellular component of cartilage, play a crucial role in maintaining cartilage homeostasis by regulating the turnover of the extracellular matrix. This balance relies on the tightly controlled processes of chondrocyte proliferation, differentiation, and apoptosis. Apoptosis is prominent in OA cartilage, with the proportion of apoptotic chondrocytes varying between less than 1% and approximately 20%, depending on the stage and severity of the disease. Importantly, the degree of chondrocyte apoptosis correlates positively with the extent of cartilage degeneration, further emphasizing its central role in OA progression. Inflammatory mediators such as ROS, nitric oxide (NO), IL-1β, TNF-α, and Fas ligand contribute to the induction of chondrocyte apoptosis in OA. Several signaling pathways are implicated in OA pathogenesis, including the NF-κB pathway, Wnt signaling, and the Notch pathway. These pathways exhibit complex, often biphasic roles in chondrocyte fate determination. For instance, depending on contextual factors, these pathways may either promote or suppress chondrocyte apoptosis and extracellular matrix degradation. Dysregulated activation of these pathways contributes to the progressive nature of OA by exacerbating inflammatory and catabolic processes. Programmed cell death, while essential for maintaining cellular homeostasis, plays complex and context-dependent roles in disease settings. In the CNS, apoptotic mechanisms may shift from protective to pathological, aggravating neuroinflammation and tissue damage. Similarly, in OA, the misregulation of chondrocyte apoptosis and associated signaling pathways underscores their contributions to disease severity. Further elucidation of these pathways could open avenues for targeted therapeutic interventions aimed at modulating PCD to preserve tissue integrity and mitigate disease progression. |
|  | f) | The role of chemokines in mediating the immune response to viral infections in the CNS is largely centered on their ability to recruit and activate antigen-specific lymphocytes. Early after viral infection, chemokines are rapidly expressed and act as key regulators of the innate immune response, serving to coordinate cellular defenses against invading pathogens. For example, the chemokine CXCL10 plays a crucial role in the CNS defense against coronavirus infections. Its expression amplifies innate immune responses, curtails disease progression, and significantly improves survival rates. This protective effect is predominantly attributed to the enhanced recruitment and activation of natural killer (NK) cells within the CNS. These NK cells facilitate viral clearance by reducing viral titers through mechanisms dependent on interferon-gamma (IFN-γ) secretion, thereby emphasizing the importance of CXCL10 in antiviral immunity. Beyond its interaction with NK cells, chemokine ligand 3 (CCL3) is instrumental in shaping the host's adaptive immune response following VICNS. During mouse hepatitis virus (MHV) infection, CCL3 is essential for the activation and function of dendritic cells (DCs), particularly the CD11c+CD11b+CD8α- subset. CCL3 facilitates the maturation, activation, and migration of these DCs to cervical lymph nodes, enhancing antigen presentation and T-cell priming. Impaired activation of CD8α- DCs has been shown to reduce IFN-γ expression while increasing IL-10 production by virus-specific T cells. This suggests that CCL3's influence on DCs enhances their capacity to activate T cells, leading to a more robust and effective host response to viral infections. These findings highlight the interplay between chemokines and antigen-presenting cells in shaping antiviral immunity within the CNS. In OA, chemokines have similarly been implicated as mediators of both tissue repair and disease progression. Synovial fluid, which is enriched with chemokines, has been shown to stimulate the migration of subchondral progenitor cells. Among these chemokines, CXCL10 stands out due to its interaction with CXCR3, the chemokine receptor expressed at the highest levels on subchondral progenitor cells. This CXCL10-CXCR3 axis is believed to facilitate the recruitment of mesenchymal progenitor cells to areas of microfracture in subchondral bone, potentially contributing to tissue repair. However, the same chemokine signaling can also drive pathological processes in OA by recruiting immune cells to the synovium. During OA progression, a variety of immune cell populations are involved in processes such as cartilage damage, bone erosion, and bone resorption. NK cells and neutrophils have been identified as key contributors to OA pathogenesis, with studies showing that they are among the first immune cells to infiltrate the synovium. These cells exert pro-inflammatory and destructive effects that exacerbate joint damage. The CXCL10-CXCR3 axis plays a central role in this process, driving the recruitment and activation of NK cells and neutrophils within OA-affected synovial tissue. Elevated CXCL10 expression in the synovium further correlates with disease severity, reinforcing its pathogenic significance. Interactions between these immune cells, mediated through CXCL10-CXCR3 signaling, create a feedback loop that amplifies local inflammation and tissue degradation, ultimately accelerating OA progression. Chemokines are pivotal regulators of immune responses in both VICNS and OA pathogenesis, with their roles spanning from mobilizing innate immune defenses to modulating adaptive responses. CXCL10 emerges as a critical chemokine in both contexts, demonstrating versatility in orchestrating protective and pathogenic immune processes. In viral infections, CXCL10 supports effective antiviral defenses, while in OA, its aberrant signaling contributes to chronic inflammation and tissue destruction. Understanding the dualistic roles of chemokines offers opportunities to develop targeted therapies that either enhance their protective functions or mitigate their contribution to disease progression. Further research into chemokine signaling pathways may uncover novel interventions for a wide range of inflammatory and degenerative disorders. |
|  | g) | The CNS plays a pivotal role in regulating the skeletal system. Anatomically, sensory nerves of the skeletal system are interconnected with the hypothalamus, notably associating with the ventromedial nucleus (VMH). The VMH assumes critical importance in governing weight regulation, glucose equilibrium, emotional responses, and reproductive functions by modulating the autonomic nervous system (ANS). Moreover, the CNS exerts regulatory control over the pathological changes occurring in OA, encompassing alterations in cartilage, subchondral bone, and the synovial membrane. This regulatory influence extends beyond mere modulation of OA's biological mechanisms; our investigation indicates a genetic interrelation between the CNS and OA. While our study did not establish a genetic causative link between the IDCNS and KOA, the findings imply a suggestive positive genetic causal association between the VICNS and KOA. This underscores the potential for VICNS to contribute to the onset of KOA. Given OA's chronic degenerative nature, acute VICNS perturbations are unlikely to trigger KOA; therefore, chronic VICNS may pose a risk factor for the development of KOA. |
| 16 | Limitations |  |
|  | a) | Primarily, the research exclusively focuses on the European population, thereby constraining the extrapolation of findings to broader demographic groups. |
|  | b) | Secondly, the differentiation of VICNS into acute and chronic categories was not feasible due to the limitations in the available GWAS dataset, limiting a comprehensive stratification of this variable. |
|  | c) | Thirdly, OA manifests across multiple joints within the human body, yet the examination of KOA within this study does not encompass the entirety of OA occurrences. It is widely acknowledged that KOA represents the most clinically prevalent form of OA, accentuating the necessity for further dedicated research into KOA given its substantial clinical implications. |
| 17 | **Conclusion** | The findings of the current study highlight a suggestive positive genetic causal association between VICNS and KOA, thereby implying a potential influence of CNS pathways on the development of KOA. In contrast, no significant genetic causal relationship was observed between IDCNS and KOA, suggesting the presence of pathway-specific mechanisms within the CNS that may selectively impact OA processes. These results provide robust genetic evidence supporting the involvement of CNS mechanisms in the regulation of OA pathophysiology. The identification of a CNS-OA axis enriches our understanding of OA as a complex, multi-system disease rather than a localized joint disorder. Importantly, this perspective underscores the critical role of neural regulatory pathways in shaping the onset and progression of OA. From a clinical standpoint, these findings advocate for a paradigm shift in OA management. Therapeutic strategies should not solely focus on the biomechanical and inflammatory dimensions of OA but also consider the central regulatory mechanisms within the CNS. Such a holistic approach could pave the way for novel interventions targeting CNS pathways, ultimately contributing to improved disease management and patient outcomes. |
|  | **Other information** |  |
| 18 | Conflict of Interest | There is no conflict of interest between all authors of this article. |
| 19 | Funding | This work was financially supported by the Scientific research and innovation platform for intelligent and precise treatment of bone and joint diseases in Shaanxi Province (No. 2024PT-13). |
| 20 | Data availability | This study utilized publicly available datasets, which were obtained from the FinnGen consortium (https://www.finngen.fi/) and IEU OpenGWAS database (https://gwas.mrcieu.ac.uk/). |

**Supplementary Table 2:** The data used in this study.

| **GWAS ID** | **Trait** | **Cases** | **Controls** | **SNPs** | **Population** |
| --- | --- | --- | --- | --- | --- |
| finn-b-AB1_VIRAL_CNS | Viral infections of the central nervous system | 1,155 | 217,637 | 16,380,466 | European |
| finn-b-G6_NEUINFL | Inflammatory diseases of the central nervous system | 1,307 | 217,485 | 16,380,466 | European |
| ebi-a-GCST007090 | Knee osteoarthritis | 24,955 | 378,169 | 29,999,696 | European |

**Supplementary Table 3:** The instrumental variables used in MR analysis between viral infections of the central nervous system and knee osteoarthritis.

|  | **SNP** | **beta.exposure** | **eaf.exposure** | **se.exposure** | **pval.exposure** | **pval.outcome** | **palindromic** | **F** |
| --- | --- | --- | --- | --- | --- | --- | --- | --- |
| 1 | rs1030108 | 0.2616 | 0.8186 | 0.0553 | 2.21E-06 | 0.461 | FALSE | 22.378 |
| 2 | rs116240632 | 0.8411 | 0.017 | 0.1778 | 2.23E-06 | 0.0935 | FALSE | 22.37834 |
| 3 | rs12653315 | -0.2399 | 0.2067 | 0.0525 | 4.80E-06 | 0.01049 | FALSE | 20.88036 |
| 4 | rs234146 | -0.3084 | 0.8861 | 0.0672 | 4.48E-06 | 0.5396 | FALSE | 21.06135 |
| 5 | rs4693205 | 0.2217 | 0.2715 | 0.0476 | 3.23E-06 | 0.8948 | FALSE | 21.6927 |
| 6 | rs6445612 | 0.2208 | 0.295 | 0.0464 | 1.98E-06 | 0.3513 | FALSE | 22.64426 |
| 7 | rs73573352 | 0.4511 | 0.05786 | 0.0932 | 1.30E-06 | 0.4445 | FALSE | 23.42663 |
| 8 | rs75944472 | 0.7309 | 0.02125 | 0.1594 | 4.53E-06 | 0.7047 | FALSE | 21.02497 |
| 9 | rs77492170 | 0.5153 | 0.04334 | 0.1097 | 2.65E-06 | 0.1233 | FALSE | 22.06496 |
| 10 | rs80249913 | 0.3878 | 0.07376 | 0.0827 | 2.72E-06 | 0.3661 | FALSE | 21.98875 |
| 11 | rs9986825 | 0.2037 | 0.3637 | 0.044 | 3.68E-06 | 0.8278 | FALSE | 21.4325 |

**Supplementary Table 4:** The instrumental variables used in MR analysis between inflammatory diseases of the central nervous system and knee osteoarthritis.

|  | **SNP** | **beta.exposure** | **eaf.exposure** | **se.exposure** | **pval.exposure** | **pval.outcome** | **palindromic** | **F** |
| --- | --- | --- | --- | --- | --- | --- | --- | --- |
| 1 | rs1030108 | 0.1895 | 0.4183 | 0.0402 | 2.39E-06 | 0.9769 | FALSE | 22.22094 |
| 2 | rs116240632 | 0.5312 | 0.03408 | 0.1128 | 2.49E-06 | 0.087231 | FALSE | 22.17655 |
| 3 | rs12653315 | 0.3128 | 0.1019 | 0.0669 | 2.96E-06 | 0.1708 | FALSE | 21.86137 |
| 4 | rs234146 | 0.7857 | 0.01713 | 0.164 | 1.65E-06 | 0.5144 | FALSE | 22.95207 |
| 5 | rs4693205 | 0.2562 | 0.1624 | 0.0543 | 2.33E-06 | 0.1546 | FALSE | 22.26151 |
| 6 | rs6445612 | 0.3238 | 0.09485 | 0.0685 | 2.25E-06 | 0.001502 | FALSE | 22.34439 |
| 7 | rs73573352 | 0.3525 | 0.07338 | 0.0769 | 4.52E-06 | 0.004028 | FALSE | 21.01172 |
| 8 | rs75944472 | -0.2603 | 0.8395 | 0.0548 | 2.04E-06 | 0.1697 | FALSE | 22.56229 |
